# Demographic, jurisdictional, and spatial effects on social distancing in the United States during the COVID-19 pandemic

**DOI:** 10.1371/journal.pone.0239572

**Published:** 2020-09-22

**Authors:** Rajesh P. Narayanan, James Nordlund, R. Kelley Pace, Dimuthu Ratnadiwakara

**Affiliations:** E.J. Ourso College of Business Administration, Louisiana State University, Baton Rouge, Louisiana, United States of America; Stony Brook University, Graduate Program in Public Health, UNITED STATES

## Abstract

Social distancing, a non-pharmaceutical tactic aimed at reducing the spread of COVID-19, can arise because individuals voluntarily distance from others to avoid contracting the disease. Alternatively, it can arise because of jurisdictional restrictions imposed by local authorities. We run reduced form models of social distancing as a function of county-level exogenous demographic variables and jurisdictional fixed effects for 49 states to assess the relative contributions of demographic and jurisdictional effects in explaining social distancing behavior. To allow for possible spatial aspects of a contagious disease, we also model the spillovers associated with demographic variables in surrounding counties as well as allow for disturbances that depend upon those in surrounding counties. We run our models weekly and examine the evolution of the estimated coefficients over time since the onset of the COVID-19 pandemic in the United States. These estimated coefficients express the revealed preferences of individuals who were able to and chose to stay at home to avoid the disease. Stay-at-home behavior measured using cell phone tracking data exhibits considerable cross-sectional variation, increasing over nine-fold from the end of January 2020 to the end of March 2020, and then decreasing by about 50% through mid-June 2020. Our estimation results show that demographic exogenous variables explain substantially more of this variation than predictions from jurisdictional fixed effects. Moreover, the explanations from demographic exogenous variables and jurisdictional fixed effects show an evolving correlation over the sample period, initially partially offsetting, and eventually reinforcing each other. Furthermore, the predicted social distance from demographic exogenous variables shows substantial spatial autoregressive dependence, indicating clustering in social distancing behavior. The increased variance of stay-at-home behavior coupled with the high level of spatial dependence can result in relatively intense hotspots and coldspots of social distance, which has implications for disease spread and mitigation.

## Introduction

The transmission of an infectious disease such as COVID-19 depends on its basic reproduction number *R*_0_, the expected number of cases directly generated from contact with an infected person. Social distancing, a non-pharmaceutical tactic, can reduce *R*_0_ by limiting contact with infected persons. Social distancing may arise naturally if individuals prefer staying at home to avoid infection and have the capacity to stay at home. Or it can arise by governmental *fiat* as a non-pharmaceutical intervention to curb the spread of the disease through restrictions on individual activities over a jurisdiction. In this paper, we examine how these factors explain social distancing using U.S. cell phone tracking data. We also examine whether individual behaviors differ across jurisdictions or exhibit other spatial patterns.

Modeling social distancing requires confronting two issues. First, social distancing depends upon the prevalence of COVID-19, and the spread of COVID-19 depends upon social distancing. Therefore, social distancing and the spread of COVID-19 are simultaneously determined, which complicates estimation of their individual effects [1, p. 151]. Second, the various ways individual jurisdictions track the disease may introduce measurement error issues that further complicate identification [1, p. 134]. Insofar as the process of obtaining a test may depend upon demographic variables such as population density (rural versus urban), age, education, race, and income, this can lead to measurement error correlated with variables in the model. For example, drive-through testing has become popular, but this may select against poorer residents without a car. Measurement error correlated with variables in the model is termed *differential measurement error*, and this can lead to bias in estimation [2].

To avoid the issues of simultaneity and measurement error, we focus on reduced form modeling of social distancing as gauged by the change in the log of the percentage of individuals staying at home each week using cell phone tracking data. The reduced form involves a regression of the dependent variable as a function of exogenous demographic variables (population density, age, education, race, and income) as well as jurisdictional fixed effects for 49 states (Hawaii excluded due to data coverage). Because of the spatial nature of a contagious disease, we also model the spillovers associated with demographic variables in surrounding counties, as well as allow for spatially dependent disturbances at the county level. This also mirrors the possible spatial aspects of natural disasters [3, 4]. We run a separate reduced form regression for each week of data and examine the evolution of the estimated coefficients over time since the onset of the COVID-19 pandemic in the United States. These estimated coefficients express the revealed preferences of individuals who chose to stay at home to avoid the disease, subject to their constraints.

The estimation results show that predicted social distance from demographic exogenous variables explains substantially more variation in individual stay-at-home behavior than predictions from jurisdictional fixed effects. In addition, the explanations from demographic exogenous variables and jurisdictional fixed effects show an evolving correlation over the sample period. For the 12th week of the data (corresponding to the second full week of April), when all states had restrictions in place following the national emergency declaration, the predictions from the demographic variables explain 43.83% of the variance while the fixed effect predictions explain 13.11% of the variance, with a correlation between these two predictive components of −0.0130. By the last week of the data in our sample (the second week of June), demographic variable predictions explain 33.18% of the variance while fixed effect predictions explain 8.83% of the variance, with a correlation between these two predictive components of 0.1778. A negative correlation implies that predictions from fixed effects and demographic effects partially cancel or offset. A zero correlation implies that the fixed effect and demographic effect predictions operate independently. A positive correlation implies that fixed effect and demographic effect predictions reinforce or complement each other.

Regression models on spatial data often result in clustered residuals or spatial error dependence. For example, consultation among officials of nearby counties could lead to them adopting similar policies and thus obtaining similar results. Indeed, we find statistically significant spatial error dependence. In contrast, we do not find statistically significant spillovers from the demographic exogenous variables in surrounding counties. In other words, the racial composition or average income of surrounding counties do not appear to significantly affect the observed social distance in a county. In addition, because the underlying exogenous demographic variables and thus the predictions show substantial spatial autoregressive dependence (between 0.851 to 0.914, depending on the week), this indicates that social distances will also cluster. Over the course of the data, the cross-sectional variation in social distances increased over nine-fold from 0.0050 in week 1 to 0.0474 in week 9, and then fell by about half to 0.0234 in week 20. The increased variance coupled with the high level of spatial dependence can result in relatively intense hotspots and coldspots of social distances.

An emerging literature surveyed by [5] and [6] points out that the constant basic reproduction number assumed in compartmental models (the Susceptible-Infectious-Recovered, or SIR models) used in epidemiology to model disease transmission is untenable given that behavioral responses to the fear of infection [7–12], underlying health conditions [13, 14], and work environment [15–18] all influence the decision to social distance. Our results show that individual behavioral responses matter, even when jurisdictional restrictions are in place, in affecting the basic reproduction number.

Models that allow for the basic reproduction number to vary over time to capture changes in behavior or policy such as [19] show a better fit with the data on the spread of COVID-19. Our results add to this line of work by suggesting that individuals with the capacity and desire to avoid risk in hard hit areas may lead to models overestimating the spread of disease based on initial infections. Conversely, individuals in areas without much incidence of the disease may not engage in social distancing and thus be quite susceptible to the disease when it finally spreads. They also suggest that the worst cases for disease spread may exist in areas where the residents have reduced capacity to avoid risk through voluntary distancing such as in poor, densely populated neighborhoods. In this setting the constant *R*_0_ assumption may hold.

## Data and background

Demographic data is collected from the American Community Survey (ACS) data provided by the U.S. Census. We use the 5-year estimates that conclude in the year 2018, because this is the most recent ACS data that includes coverage for every U.S. county. We take a county’s population size to be the population estimated by the U.S. Census for July 1, 2019. To convert the population level into a population density, we obtain county land area from the 2019 release of the U.S. Census Gazetteer data.

Table 1 reports summary statistics of the exogenous variables used in this study. Population density (Pop) is the most highly skewed variable, with a minimum of 0.04 people per square mile (Yukon-Koyukuk, AK) and a maximum of 71,872.6 people per square mile (New York, NY). In the median county, 29.3 percent of households have at least one child (Child) under 18 years of age. In the median county, 87 percent of the population have not obtained a bachelor’s degree or higher (HS), and 89.9 percent of the population is white (White). The median household income (Income) for the median county is $49,871.8.

**Table 1:** Percentiles of exogenous demographic variables.

Percentiles	Pop	Child	HS	White	Income
Min	0.0	6.7	52.0	3.9	20,188.0
5th	1.0	20.7	75.2	47.5	33,528.2
10th	4.0	22.8	78.3	60.6	36,876.4
25th	16.3	26.0	83.0	77.3	42,479.5
50th	44.4	29.3	87.0	89.9	49,871.8
75th	114.5	32.5	90.1	95.1	57,468.2
90th	396.0	36.2	92.1	96.9	67,184.6
95th	856.0	38.0	93.2	97.6	77,227.2
Max	71,872.6	70.1	100.0	100.0	136,267.0

We infer county-level rates of individuals staying at home using GPS data from SafeGraph. The company collects anonymized location data of individuals via GPS pings from cell phones. In response to the pandemic, SafeGraph released their aggregated and anonymized data to researchers, and began publishing additional data, including a Social Distancing Metrics dataset. This dataset covers all 50 U.S. states as well as Washington, D.C. We find incomplete county coverage in Hawaii, and therefore exclude it from our analysis. The Social Distancing Metrics data record, aggregated to a Census Block Group (CBG) level, the number of active devices in the CBG on a given day and the count of those devices that never leave the user’s inferred home location on that day. We aggregate these CBG-level numbers to the county-level and then define our proxy for social distancing by dividing the number of devices completely at home by the total number of active devices. The average rate of devices staying at home over a given week is then taken to be the level of social distancing in the county.

Table 2 presents the evolution of this measure of social distancing over the 21 weeks of data in our sample beginning with the third week of January, 2020. The median percentage of people social distancing is about 25% prior to President Trump’s National Emergency Declaration on March 14, 2020 (week 7). Following the declaration, there is a sharp increase in individuals staying at home in weeks 8 through 12. It begins to decline in mid-April when individual states relaxed their stay-at-home restrictions, but stays elevated relative to pre-emergency levels. The variation in the range of social distancing shows a similar pattern across the weeks.

**Table 2:** Percentiles of percentage staying home by week.

Week	Min	5th	10th	25th	50th	75th	90th	95th	Max
1	6.68	17.30	18.33	20.09	22.22	24.74	27.35	29.30	50.78
2	7.37	17.19	18.16	19.94	22.16	24.56	27.24	29.59	50.11
3	8.19	19.62	20.90	23.10	25.52	28.11	30.86	32.78	50.17
4	5.54	18.25	19.42	21.39	23.58	26.07	28.45	30.22	47.79
5	5.89	18.27	19.28	21.01	23.17	25.57	27.96	30.05	48.48
6	6.58	19.80	20.81	22.43	24.57	26.66	29.11	30.90	52.05
7	8.16	16.49	17.40	18.86	20.75	22.77	24.57	26.09	49.51
8	6.54	16.53	17.76	19.65	21.68	23.80	25.83	27.16	49.74
9	6.25	23.35	24.71	27.04	29.51	32.08	35.02	37.02	50.84
10	5.15	23.37	24.92	27.64	31.04	35.22	39.11	41.38	55.73
11	7.68	28.41	29.72	32.68	35.69	39.28	42.74	45.17	59.49
12	6.01	29.09	30.50	33.00	36.23	39.94	43.39	45.77	60.51
13	6.80	26.75	28.48	31.35	34.90	38.78	42.62	45.24	59.26
14	4.80	24.64	26.14	28.84	32.09	35.73	39.84	42.42	57.42
15	5.70	23.22	24.87	27.45	30.89	34.63	38.45	40.93	55.26
16	7.71	22.66	24.23	27.40	31.40	35.31	38.83	41.61	57.80
17	5.14	22.08	23.67	26.47	29.99	33.71	37.16	39.84	53.55
18	4.50	21.04	22.55	25.23	28.72	32.01	35.52	38.05	52.48
19	3.36	20.93	22.23	24.58	27.65	31.18	34.69	36.95	54.00
20	4.25	19.66	20.88	23.08	26.09	29.72	33.52	35.61	54.81
21	9.68	20.21	21.66	23.98	27.30	30.90	34.85	36.55	53.15

The presidential emergency declaration was not the only governmental response to the pandemic. States responded to the health care crisis by issuing non-pharmaceutical interventions (NPI), such as mandated closure of certain businesses. The responses varied by state, and were implemented at varying points over our sample period. Moreover, these *de jure* NPIs have heterogeneous *de facto* implementation/enforcement. As [20] document, an extensive variety of the actual policies were implemented at different times over the outbreak. This heterogeneity impedes direct modeling of specific measures. To address these issues, we include state fixed effects along with other control variables. The efficacy of the underlying restrictions will implicitly appear as evolving parameter estimates by week.

We also obtain county-level disease data from the *New York Times* and report summary statistics of new disease counts in Table 3 for illustrative purposes. The highly decentralized nature of disease tracking leads to significant issues in U.S. disease case data. Substantial heterogeneity exists at the county level due to differences in testing eligibility (whether an individual can receive a test), testing access (whether the county had test kits available), testing rules (e.g., whether tests are required on the deceased), and reporting rules (e.g., classification of results for non-residents). As Table 3 shows, fewer than five percent of counties reported new cases in the week of the national emergency declaration. This is not because cases were rare at this time, but rather because of limitations in testing availability during the first months of the pandemic [21, 22]. The various sources of measurement error or selection mechanisms present in these data motivate the approach we use which avoids case data. We discuss the statistical justifications for this in the following section.

**Table 3:** Percentiles of new cases by week.

Week	Min	5th	10th	25th	50th	75th	90th	95th	Max
1	0.00	0.00	0.00	0.00	0.00	0.00	0.00	0.00	0.00
2	0.00	0.00	0.00	0.00	0.00	0.00	0.00	0.00	1.00
3	0.00	0.00	0.00	0.00	0.00	0.00	0.00	0.00	0.00
4	0.00	0.00	0.00	0.00	0.00	0.00	0.00	0.00	0.00
5	0.00	0.00	0.00	0.00	0.00	0.00	0.00	0.00	2.00
6	0.00	0.00	0.00	0.00	0.00	0.00	0.00	0.00	7.00
7	0.00	0.00	0.00	0.00	0.00	0.00	0.00	0.00	80.00
8	0.00	0.00	0.00	0.00	0.00	0.00	0.00	0.00	330.00
9	0.00	0.00	0.00	0.00	0.00	0.00	1.00	13.00	1,802.00
10	0.00	0.00	0.00	0.00	0.00	2.00	23.00	61.65	6,646.00
11	0.00	0.00	0.00	0.00	1.00	10.00	45.00	117.30	7,953.00
12	0.00	0.00	0.00	0.00	2.00	12.00	54.00	131.30	9,155.00
13	0.00	0.00	0.00	0.00	2.00	12.00	55.00	136.65	6,687.00
14	0.00	0.00	0.00	0.00	2.00	13.00	64.30	157.65	9,302.00
15	0.00	0.00	0.00	0.00	2.00	15.00	68.00	173.65	11,750.00
16	0.00	0.00	0.00	0.00	2.00	15.00	71.00	171.65	10,331.00
17	0.00	0.00	0.00	0.00	2.00	15.00	67.00	163.30	9,563.00
18	0.00	0.00	0.00	0.00	3.00	18.00	72.00	163.25	9,792.00
19	0.00	0.00	0.00	0.00	3.00	17.00	68.00	165.25	5,915.00
20	0.00	0.00	0.00	0.00	3.00	18.25	75.00	165.65	4,502.00
21	0.00	0.00	0.00	0.00	4.00	18.00	79.00	175.65	5,194.00

## Empirical strategy

As mentioned earlier, the virus contagiousness depends on social distancing, and social distancing may depend upon both the contagiousness and virulence of the virus. Ideally, one could model these as simultaneous equations where the system dependent variable *Y*_*t*_ is an *n* × 2 matrix. Specifically, *Y*_*t*_ contains an *n* × 1 vector of observations on the virus *v*_*t*_ and an *n* × 1 vector of observations on the social distancing variable, *h*_*t*_, which could represent the propensity of residents to stay in their homes at time *t* as shown in (1) and (2). In (2) *X* represents the exogenous explanatory variables, Γ_*t*_ represents the parameters governing the influence of each endogenous variable on the other at time *t*, and *E*_*t*_ represents the disturbances at time *t*.

$$\begin{array}{cc} \underset{\left(n\times 2\right)}{Y_{t}} & =[\begin{array}{cc} \underset{\left(n\times 1\right)}{h_{t}} & \underset{\left(n\times 1\right)}{v_{t}} \end{array}] \end{array}$$ (1)

$$\begin{array}{cc} \underset{\left(n\times 2\right)}{Y_{t}}\underset{\left(2\times 2\right)}{\Gamma_{t}} & =\underset{\left(n\times k\right)}{X_{}}\underset{\left(k\times 2\right)}{B_{t}}+\underset{\left(n\times 2\right)}{E_{t}} \end{array}$$ (2)

The equation representation in (2) is referred to as a structural form of the model [23, p. 528] and these require special techniques or data to avoid inconsistent estimation. Specifically, if the virus *v*_*t*_ and the social distancing variable *h*_*t*_ are simultaneously determined, estimating a single equation of social distancing as a function of the virus will lead to inconsistent parameter estimates of the effect of the virus *v*_*t*_ on social distancing *h*_*t*_. In other words, estimating an equation such as *h*_*t*_ = *x*_1_ *β*(*t*)_1_ + ⋯ + *x*_*p*_ *β*(*t*)_*p*_ + *v*_*t*_ *β*(*t*)_*p*+1_ + *ε*(*t*) will lead to an inconsistent parameter estimate of *β*(*t*)_*p*+1_, the effect of the virus *v*_*t*_ on social distancing *h*_*t*_ [1, p. 151]. A similar situation exists in economics where modeling supply and demand but ignoring simultaneity led to incorrect signs on the effects of price on supply and demand. This motivated many of the techniques and ideas associated with simultaneous equations from the 1930s onward [24].

However, the identification and estimation of structural equations in (2) sometimes proves challenging. The most common means of obtaining identification requires a variable (instrument) that affects one of the endogenous variables, but not the other. Unfortunately, this exogeneity condition cannot be tested empirically and becomes a potential weak point in the analysis. Moreover, the non-uniform nature of reporting of COVID-19 tests, hospitalization, and deaths across jurisdictions raises issues about measurement error concerning the virus. In a simultaneous equation system, measurement error in one equation (virus) could affect the other equation (social distancing). To avoid this, we resort to modeling the unrestricted reduced form [23, p. 528] as shown in (3). In an unrestricted reduced form each dependent variable can be estimated consistently when using all the exogenous variables in *X*. The resulting parameter estimates Π_*t*_ in (4) show the effect of a particular exogenous variable on the dependent variable.

$$\begin{array}{cc} Y_{t} & =X\Pi_{t}+E_{t}\Gamma_{t}^{-1} \end{array}$$ (3)

$$\begin{array}{cc} \Pi_{t} & =B_{t}\Gamma_{t}^{-1} \end{array}$$ (4)

Because the exogenous variables, *X*, do not change over the period, each cross-sectional estimate can yield different sets of estimates of Π_*t*_. In this way choices by individuals concerning their desire to avoid the virus can express an evolving response to the presence of the disease. Staying at home reveals a preference for safety (subject to the capacity to stay at home). Our interest concerns the changes in behavior over the rise of the disease and to do this we difference the reduced form equations at time *t* and time 0 as shown in (5).

$$\begin{array}{c} Y_{t}-Y_{0}=X[\Pi_{t}-\Pi_{0}]+\left[E_{t}\Gamma_{t}^{-1}-E_{0}\Gamma_{0}^{-1}\right] \end{array}$$ (5)

Note, for the unrestricted reduced form one can estimate this equation by equation. In this case, since the focus lies on social distancing as a function of the exogenous variables, we can move to a simpler single equation model, a subject taken up in the next section.

In summary, social distancing and disease are determined simultaneously and a simple single equation estimation that treats one of the endogenous variables as a typical right hand side variable can lead to inconsistent estimates. Although one could attempt to simultaneously model these variables using a structural form, identification of these systems can be challenging. In addition, the disease data have many non-uniform reporting issues that may adversely affect estimation. To avoid this we resort to unrestricted reduced form modeling. The repercussions of the prevalence of the disease show up in the evolving parameter estimates since the exogenous variables stay fixed over the period of the disease. By differencing the reduced forms at time 0 and time *t* we see the change in individual behavior as a function of the exogenous variables.

## Model

In this section we describe the actual single equation model used in estimation based on the reduced form interpretation in the previous section. We begin by defining *y*(*t*) as log of the percentage of the population staying at home at week *t*, *h*(*t*), as in (6). In contrast to the previous section, we switch to a parametric notation indexed by *t* because this allows use of both subscripts and powers for the various components in the models. The indexing of the parameters, disturbances, and dependent variable by *t* in the model indicates a series of cross-sectional regressions where the values of the parameters over time reflect individuals’ revealed preference for safety by staying at home. Because our interest focuses on the relative change in *y*(*t*) over time our dependent variable Δ(*t*) in (7) equals *y*(*t*) − *y*(0). Using changes may help reduce omitted variables that have a similar influence over period 0 and *t*. Because our period of differencing is relatively short (up to 20 weeks), many influences from omitted variables may be reduced.

$$\begin{array}{c} \underset{\left(n\times 1\right)}{y(t)}=\ln(h(t)) \end{array}$$ (6)

$$\begin{array}{c} \underset{\left(n\times 1\right)}{\Delta (t)}=y(t)-y(0) \end{array}$$ (7)

We selected variables to represent the basic demographic characteristics of population density, age, education, race, and income. For ease of interpretation, we selected only one variable from each major category. Specifically, the explanatory variables include the log of the population density (Pop), log of the percentage of households with children under 18 (Child), log of the percentage of workers without a college degree (HS), log of percentage White in the population (White), and log of median income (Income) as shown in (8) to (12). Because some counties have no minority residents, we coded the race variable as White to avoid taking the log of 0. Similarly, some counties have no college graduates and so we coded the education variable as the percentage of the population without a college degree. This would include individuals that did not complete high school, but for simplicity we denoted this as HS. The Child variable captures some element of age, but also captures the effect of school shutdowns which may have forced many parents to stay at home.

$$\begin{array}{c} \text{Pop}=\ln(\text{Population})-\ln(\text{Area}) \end{array}$$ (8)

$$\begin{array}{c} \text{Child}=\ln(\text{Households}\;\text{With}\;\text{Persons}\;\text{Under}\;\text{18})-\ln(\text{Population}) \end{array}$$ (9)

$$\begin{array}{c} \text{HS}=\ln(\text{People}\;\text{without}\;\text{Collge}\;\text{Degree})-\ln(\text{Population}) \end{array}$$ (10)

$$\begin{array}{c} \text{White}=\ln(\text{White}\;\text{Individuals})-\ln(\text{Population}) \end{array}$$ (11)

$$\begin{array}{c} \text{Income}=\ln(\text{Median}\;\text{Income}) \end{array}$$ (12)

We use the demographic explanatory variables in (8) to (12) to compose the *n* × 5 matrix *Z* in (13) and also create a *n* × 50 matrix *S* of dichotomous variables for 49 States in (14) as well as a constant vector (intercept). Specifically, we include a constant term *ι*_*n*_ defined in (15) and exclude the District of Columbia from the dichotomous variables to avoid perfect multicollinearity in (14).

$$\begin{array}{c} \underset{\left(n\times 5\right)}{Z}=[\begin{array}{ccccc} \text{Density} & \text{Child} & \text{HS} & \text{White} & \text{Inc} \end{array}] \end{array}$$ (13)

$$\begin{array}{c} \underset{\left(n\times 50\right)}{S}=[\begin{array}{cccccccc} \iota_{n} & \text{AL} & \text{AK} & \text{AZ} & \ldots & \text{WV} & \text{WI} & \text{WY} \end{array}] \end{array}$$ (14)

$$\begin{array}{c} \underset{\left(n\times 1\right)}{\iota_{n}}={\left[\begin{array}{cccc} 1 & 1 & \ldots & 1 \end{array}\right]}^{\prime } \end{array}$$ (15)

The contagious nature of COVID-19 suggests the need of explicitly incorporating spatial aspects into the model. We do so in four ways: (1) we examine changes in social distancing behavior and that should reduce the influence of omitted variables with relatively constant importance; (2) we incorporate fixed jurisdictional effects (*S*); (3) we allow for spillovers from exogenous demographic variables from surrounding counties to enter the model; and (4) we allow for spatial dependence in the disturbances which can capture nearby omitted variables.

To specify spillovers and dependence in the disturbances, spatial econometrics often uses a *n* × *n* weight matrix *W* to create spatial lags of variables so that *Wv* becomes the spatial lag of an *n* × 1 variable *v*. This matrix contains positive, fixed elements if observations neighbor each another and zero elements for non-neighboring observations as shown in (16). Also, to prevent neighbors from predicting themselves the diagonal elements of *W* equal 0 as specified in (17). Most commonly, *W* is row normalized so that each row sums to 1 as in (18). Because each row sums to 1 and *W* contains only non-negative elements, *W* is a row-stochastic matrix [25, 26]. Note, a matrix can be row-stochastic in the linear algebra sense while containing only non-stochastic elements in the probabilistic sense. Therefore, *Wv* becomes an average of observations that lie nearby each element of *v* and (*Wv*)_*i*_ does not contain *v*_*i*_. Having λ(*t*)∈(−1, 1) as shown in (18) leads to a sufficient condition for a positive definite covariance matrix in many spatial econometric models using row-stochastic *W*.

$$\begin{array}{c} W_{ij}>0\;\text{if}\;\text{observations}\;i,j\;\text{are}\;\text{neighbors,}\;\text{otherwise}\;W_{ij}=0 \end{array}$$ (16)

$$\begin{array}{c} W_{ii}=0 \end{array}$$ (17)

$$\begin{array}{c} W\iota_{n}=\iota_{n},\;\lambda \left(t\right)\in \left(-1,1\right) \end{array}$$ (18)

To incorporate all of these spatial aspects into a model, we selected the spatial Durbin model (SDM) in (19) and (20) as well as the spatial Durbin error model (SDEM) with jurisdictional fixed effects as general specifications as shown in (21) and (22). The SDM and SDEM subsume non-spatial ordinary least squares (OLS) with and without jurisdictional fixed effects, the spatial lag of *X* model (SLX) with and without jurisdictional fixed effects, and the spatial error model (SEM) with and without jurisdictional fixed effects. However, they do not nest each other and the SDM also nests the spatial autoregressive model (SAR). Note, the acronymn SAR stands for a spatial error model in spatial statistics such as in Orr (1975), but stands for a spatially lagged dependent variable model in spatial econometrics. The SDM can yield models with global spatial spillovers while the SDEM only yields local spatial spillovers. For untransformed dependent and independent explanatory variables, the estimates from OLS, SLX, SEM, and SDEM also equal the respective marginal effects. For the SDM and SAR the parameter estimates do not equal the marginal effects. For a discussion of these two models and the restrictions that lead to OLS, SLX, and SEM see [26], [27], as well as [28]. Note, other research using these spatial econometric models for investigating the virus, but not social distance, has found significant spatial influences on the disease [29, 30].

$$\begin{array}{c} \Delta (t)=\lambda \left(t\right)W\Delta \left(t\right)+S\cdot \alpha \left(t\right)+Z\cdot \beta {\left(t\right)}_{SDM}+W\;Z\cdot \theta {\left(t\right)}_{SDM}+\varepsilon \left(t\right) \end{array}$$ (19)

$$\begin{array}{c} \varepsilon (t)∼N(0,\sigma^{2}I_{n}) \end{array}$$ (20)

$$\begin{array}{c} \Delta (t)=S\cdot \alpha \left(t\right)+Z\cdot \beta {\left(t\right)}_{SDEM}+W\;Z\cdot \theta {\left(t\right)}_{SDEM}+\xi \left(t\right) \end{array}$$ (21)

$$\begin{array}{c} \xi (t)=\lambda (t)W\xi (t)+\varepsilon (t) \end{array}$$ (22)

To show the ability of the SDEM to nest other well known spatial models in more detail, we present these more specific models in (23) to (28). The SDEM with fixed effects subsumes the non-spatial OLS with and without jurisdictional fixed effects (23, 24), spatial lag of *X* (SLX) with and without jurisdictional fixed effects (25, 26), and the spatial error model (SEM) with and without jurisdictional fixed effects (27, 28). In the models without jurisdictional fixed effects, the scalar *κ*(*t*) represents the effect at time *t* of the constant vector *ι*_*n*_ or intercept.

$$\begin{array}{c} \Delta (t)=\iota_{n}\underset{\left(1\times 1\right)}{\kappa (t)}+Z\beta {\left(t\right)}_{O}+\varepsilon \left(t\right) \end{array}$$ (23)

$$\begin{array}{c} \Delta (t)=S\alpha \left(t\right)+Z\beta {\left(t\right)}_{O-FE}+\varepsilon \left(t\right) \end{array}$$ (24)

$$\begin{array}{c} \Delta (t)=\iota_{n}\kappa \left(t\right)+Z\beta {\left(t\right)}_{SLX}+W\;Z\theta {\left(t\right)}_{SLX}+\varepsilon \left(t\right) \end{array}$$ (25)

$$\begin{array}{c} \Delta (t)=S\alpha \left(t\right)+Z\beta {\left(t\right)}_{SLX-FE}+W\;Z\theta {\left(t\right)}_{SLX-FE}+\varepsilon \left(t\right) \end{array}$$ (26)

$$\begin{array}{c} \Delta (t)=\iota_{n}\kappa \left(t\right)+Z\beta {\left(t\right)}_{SEM}+\xi \left(t\right) \end{array}$$ (27)

$$\begin{array}{c} \Delta (t)=S\alpha \left(t\right)+Z\beta {\left(t\right)}_{SEM-FE}+\xi \left(t\right) \end{array}$$ (28)

Note, if *θ*(*t*) is non-zero and *Z* and *WZ* exhibit correlation, estimates of *β*(*t*) in a model without *WX* can exhibit bias [26, 28]. If λ(*t*) ≠ 0, inference based on the assumptions of independent disturbances can be inconsistent [26, 31]. Estimation of the independent error models in (23) to (26) can use ordinary least squares. Estimation of the SDM, SDEM and the SEM in (19), (21), (27) and (28) requires other techniques such as maximum likelihood [26, 31]. Note, minimizing sum of squared errors will typically lead to overly large values of λ(*t*), whereas maximum likelihood involves a log-determinant term ln|*I*_*n*_ − λ(*t*)*W*| that penalizes such large values [26, 31]. We used maximum likelihood to produce the estimates in the empirical section.

In terms of interpretation, *β*(*t*) from the non-spatial OLS and SEM has the usual partial derivative interpretation where changes to an explanatory variable’s value for an observation only affect that observation’s dependent variable. However, the SLX and SDEM contain the term *WZ* and this allows for an observations’ neighbor value to have an effect on the observation. In the SLX and SDEM *β*(*t*) measures the *direct* effects and *θ*(*t*) measures the *indirect* effects of a neighbor on an observation [26, 27]. Because of the double log specification of the empirical model, one can interpret the estimates as elasticities.

Various specifications such as the spatially autoregressive model (SAR) or the spatial Durbin model (SDM) incorporate a spatially lagged dependent variable, *W*Δ(*t*).

$$\begin{array}{c} \Delta (t)=\lambda \left(t\right)W\Delta \left(t\right)+\iota_{n}\kappa (t)+Z\beta {\left(t\right)}_{SAR}+\varepsilon \left(t\right) \end{array}$$ (29)

$$\begin{array}{c} \Delta (t)=\lambda \left(t\right)W\Delta \left(t\right)+S\alpha \left(t\right)+Z\beta {\left(t\right)}_{SAR-FE}+\varepsilon \left(t\right) \end{array}$$ (30)

$$\begin{array}{c} \Delta (t)=\lambda \left(t\right)W\Delta \left(t\right)+\iota_{n}\kappa \left(t\right)+Z\beta {\left(t\right)}_{SDM}+W\;Z\theta {\left(t\right)}_{SDM}+\varepsilon \left(t\right) \end{array}$$ (31)

$$\begin{array}{c} \Delta (t)=\lambda \left(t\right)W\Delta \left(t\right)+S\alpha \left(t\right)+Z\beta {\left(t\right)}_{SDM-FE}+W\;Z\theta {\left(t\right)}_{SDM-FE}+\varepsilon \left(t\right) \end{array}$$ (32)

The incorporation of the spatially lagged dependent variable for these models means that the estimates do not equal the marginal effects in contrast to the error models (SLX, SEM, and SDEM) presented earlier. To use the example of SDM with fixed effects from (32), solving for Δ(*t*) yields an expression in (33) with the endogenous variable on the left-hand side and the exogenous variables on the right-hand side. Note, (*I*_*n*_ − λ(*t*)*W*)^−1^ = *I*_*n*_+ λ(*t*)*W*+ λ(*t*)^2^ *W*^2^+ … and thus this has the effect of incorporating the immediate spatial lag of the exogenous variables, a spatial second order lag of the exogenous variables, and so forth. Terms such as *W*^2^ provide a second-order spatial lag so that *W*^2^ *v* equals *W*(*Wv*) so that this term gives the spatial average of the spatial average of *v*. Higher order powers of *W* act in a similar way. A high order power of *W* will often have all positive elements and therefore each observation affects every other observation and this means that models using such high powers of *W* have a global nature. Therefore, the SDM contains both local and global spatial lags of the exogenous variables. Note that if the magnitude of λ(*t*) is low, (*I*_*n*_ − λ(*t*)*W*)^−1^ might converge in just a few terms and therefore the model still has a local nature. This sometimes makes it difficult to distinguish SEM and SAR or SDEM and SDM when low levels of spatial dependence exist.

$$\begin{array}{c} \Delta (t)={\left(I_{n}-\lambda \left(t\right)W\right)}^{-1}\left[S\alpha \left(t\right)+Z\beta {\left(t\right)}_{SDM}+W\;Z\theta {\left(t\right)}_{SDM-FE}+\varepsilon \left(t\right)\right] \end{array}$$ (33)

To summarize, we use differencing of the dependent variable, jurisdictional fixed effects, demographic variables, spatial spillovers, and spatial error dependence to explain social distancing. First, differencing of the dependent variable may reduce the influence of any omitted variable whose importance does not change much over the sample time period (20 weeks). Second, since states have set forth restrictions due to the disease, jurisdictional fixed effects should play a role in social distancing. Third, most social cross-sectional phenomena vary with demographics and thus the inclusion of demographic exogenous variables. Specifically, the nature of the disease suggests that population density matters. Also, having a child present in the household may require an adult in residence with school and day care closures. Education often dictates the types of jobs that individuals hold and many of the jobs that allow remote work require more education. Income also allows individuals the ability to socially isolate. Fourth, a contagious disease suggests spatial spillovers and so these should be present in the more general models. Fifth, cross-sectional data models often exhibit spatially dependent residuals and so a model should be capable of handling them. This still leaves a number of possible specifications. Given the array of possible spatial specifications, we wish to examine their performance in the next section. We also examine a number of alternative specifications in a later section in which we explore the robustness of our principal model.

## Specification search

Because the choice of the model as well as the form of *W* has potential to affect the results, we conducted a specification search using the well known Bayesian Information Criterion [32] or BIC. For the BIC, the optimal model has the lowest value out of a series of candidate models. Specifically, we examined SAR, SEM, SDEM, and SDM with and without jurisdictional fixed effects. The results appear in Table 4 (note the BIC that we report is scaled by *n* to facilitate formatting). For the models with fixed effects, during the first five weeks, SAR had the lowest BIC, during week six SDM had the lowest BIC, and during weeks 7–20 SEM displayed the lowest BIC. For the models using only a constant, SEM showed the lowest BIC for weeks 1–7, 12, and 14–20, SDM showed the lowest BIC from weeks 8–11 and 13, while SDEM showed the lowest BIC in week 12. Taken as a whole the SEM seemed to display the lowest levels of BIC across the weeks and so we will take this as our base model. Note, the models without fixed effects provided a lower BIC than the models with fixed effects. However, the role of the fixed effects is theoretically important in this application and so we will leave these in our base model. We also examined different numbers of neighbors. Using 15 nearest neighbors resulted in the lowest BIC in week 20 for SEM with fixed effects (3.7288). Using 14, 16 nearest neighbors yielded higher values of 3.7294, 3.7304. In summary, we will use SEM with fixed effects (SEM-FE) with 15 nearest neighbors as our base model.

**Table 4:** BIC across models across weeks (15 nearest neighbors).

Week	SEM	SDEM	SDM	SAR	SEM	SDEM	SDM	SAR
	FE	FE	FE	FE	Con.	Con.	Con.	Con.
1	2.5518	2.5613	2.5504	2.5396	2.4607	2.4726	2.4726	2.4632
2	3.0407	3.0487	3.0388	3.0292	2.9404	2.9505	2.9499	2.9436
3	3.0842	3.0933	3.0855	3.0757	3.0078	3.0207	3.0204	3.0093
4	3.0608	3.0697	3.0599	3.0503	2.9878	2.9967	2.9962	2.9925
5	3.1429	3.1554	3.1499	3.1396	3.0696	3.0751	3.0744	3.0978
6	3.8823	3.8858	3.8783	3.8837	3.7998	3.8031	3.8012	3.8142
7	4.2429	4.2515	4.2472	4.2469	4.1638	4.1728	4.1714	4.1834
8	4.2248	4.2274	4.2251	4.2361	4.1681	4.1638	4.1618	4.2309
9	4.2628	4.2659	4.2635	4.2722	4.2054	4.2023	4.2018	4.2596
10	3.8942	3.8992	3.8956	3.9120	3.8360	3.8364	3.8354	3.9218
11	3.8481	3.8537	3.8489	3.8619	3.7873	3.7840	3.7830	3.8823
12	3.9223	3.9296	3.9249	3.9367	3.8579	3.8611	3.8615	3.9380
13	3.9104	3.9175	3.9130	3.9279	3.8354	3.8330	3.8328	3.9043
14	3.7966	3.8072	3.8049	3.8135	3.7241	3.7288	3.7288	3.7785
15	3.7573	3.7699	3.7669	3.7692	3.6913	3.7001	3.6993	3.7389
16	3.7505	3.7621	3.7592	3.7598	3.6918	3.7016	3.7013	3.7430
17	3.7297	3.7417	3.7357	3.7363	3.6618	3.6726	3.6716	3.6949
18	3.7037	3.7148	3.7123	3.7084	3.6367	3.6458	3.6452	3.6613
19	3.7328	3.7436	3.7407	3.7401	3.6657	3.6745	3.6737	3.6913
20	3.7288	3.7395	3.7352	3.7373	3.6526	3.6616	3.6611	3.6768

The various information criteria such as Akaike Information Criterion (AIC) [33, 34] and BIC differ on the weight given to parsimony versus performance and a criterion such as AIC which does not penalize model complexity as much as the BIC would result in different rankings of the models. To further examine the performance of alternative models, in a later section we fit the SAR, SEM, SDEM, and SDM with and without jurisdictional fixed effects, SEM FE with 10 as well as 20 nearest neighbors, and SEM FE applied to an alternate measure of social distance. We subsequently examine the correlation among the marginal effects of these 8 specifications across the 20 weeks of the data. In the next section, we provide our base model SEM-FE estimates.

## Estimates

We use the spatial error model (SEM) with fixed effects for each jurisdiction as the baseline model as described previously in (28) and (22). Estimation uses a *W* matrix based on 15 nearest neighbors. We examine the sensitivity to choices of *W* in the following section. The results of estimation of the demographic exogenous variables appear in Table 5 where *t*-values appear below their respective estimates. The results of estimation of the significant fixed effect coefficients appear in Table 6.

**Table 5:** ‘stay-at-home’ SEM estimates (with state fixed effects—Not shown).

Week	Pop	Child	HS	White	Inc	λ(*t*)
1	−0.007	−0.021	−0.068	−0.006	−0.007	0.542
	−6.286	−2.761	−2.404	−0.971	−0.758	14.116
2	−0.007	−0.009	−0.157	0.005	−0.000	0.657
	−4.388	−0.908	−4.338	0.634	−0.021	18.708
3	−0.005	0.000	−0.151	0.003	−0.004	0.375
	−3.435	0.046	−4.088	0.367	−0.381	8.212
4	−0.008	0.014	−0.177	0.016	0.009	0.465
	−5.354	1.473	−4.845	2.130	0.760	11.089
5	−0.014	0.038	−0.340	0.057	0.011	0.429
	−8.856	3.848	−8.892	7.340	0.909	10.131
6	0.014	0.020	−0.119	0.021	0.037	0.442
	5.986	1.365	−2.164	1.876	2.207	10.170
7	0.029	0.068	−0.141	0.021	0.077	0.351
	10.988	3.962	−2.151	1.627	3.866	7.787
8	0.032	0.119	−0.442	0.074	0.212	0.423
	11.861	6.938	−6.758	5.582	10.518	9.521
9	0.042	0.125	−0.455	0.089	0.240	0.367
	15.349	7.145	−6.835	6.680	11.744	8.053
10	0.027	0.100	−0.653	0.072	0.191	0.439
	11.762	6.855	−11.669	6.363	11.091	9.944
11	0.026	0.101	−0.721	0.078	0.173	0.420
	11.680	7.046	−13.150	7.118	10.311	9.455
12	0.025	0.094	−0.797	0.069	0.179	0.407
	10.930	6.359	−13.997	6.123	10.342	9.041
13	0.030	0.090	−0.769	0.065	0.163	0.502
	13.012	6.090	−13.555	5.657	9.320	11.932
14	0.028	0.078	−0.874	0.024	0.141	0.455
	12.872	5.619	−16.233	2.194	8.646	10.754
15	0.026	0.060	−0.954	0.019	0.104	0.409
	12.391	4.449	−18.003	1.865	6.551	9.377
16	0.026	0.066	−0.911	0.002	0.117	0.377
	12.536	4.922	−17.314	0.176	7.421	8.424
17	0.027	0.065	−0.828	−0.008	0.091	0.432
	12.733	4.854	−15.889	−0.791	5.768	10.074
18	0.029	0.061	−0.747	−0.002	0.083	0.349
	14.031	4.606	−14.650	−0.230	5.437	7.675
19	0.025	0.071	−0.822	−0.022	0.060	0.416
	12.149	5.304	−15.784	−2.179	3.855	9.674
20	0.025	0.046	−0.768	−0.011	0.046	0.399
	12.190	3.393	−14.786	−1.030	2.925	9.018

**Table 6:** State fixed effect estimates from reduced form SEM significant at 1% level.

Week	9	10	11	12	13
C.	−1.47	.	.	1.13	1.13
AL	.	0.32	0.34	.	.
GA	.	0.34	0.32	.	.
LA	0.39	0.34	0.30	.	.
MI	.	0.31	0.31	0.32	.
MS	.	0.35	0.36	.	.
NJ	.	0.32	.	0.32	0.33
OK	.	0.35	.	.	.
TX	.	0.35	.	.	.

Over the course of time the estimated coefficient on population density (Pop) started at a significant −0.007 in week 1 indicating that urban dwellers stayed home less than rural dwellers at the beginning of the rise of the disease. However, this changed over time until population density showed a positive 0.025 coefficient indicating that urban dwellers stayed home more than rural dwellers by week 20. The break in behavior occurred between weeks 5 and 6 where population density switched from a significant −0.014 to a significant 0.014. Percentage of households with children (Child) also showed an evolution from significant −0.021 to 0.046, with a large break occurring from week 6 to week 8. This may represent the repercussions of school closures in many areas. The Child variable coefficient reached a peak of 0.125 in week 9 and declined to 0.046 in week 20. The coefficient associated with the log of the proportion of individuals without a college degree (HS) went from a −0.068 to a peak of −0.954 in week 15, with a break happening between week 7 and 8. The coefficient declined to a significant −0.768 by week 20. This could have happened because individuals with a college degree may have been able to work at home which increased the percentage of the time they stayed at home relative to their less-educated counterparts. Areas with a higher log proportion of whites (White) showed an insignificant effect of staying at home in week 1 of −0.006, which rose to a significant 0.089 by week 9 and declined back to insignificance (−0.011) by week 20. Again, the largest break happened between weeks 7 and 8. Finally, median income (Inc) also began with an insignificant effect in week 1 of −0.007, then rose by a factor of 22 by week 13 to a significant 0.502, again with a large break between weeks 7 and 8. From the peak it declined in magnitude to a significant 0.046 in week 20. Note, estimates of the spatial error dependence parameter λ(*t*) always exhibited significance but generally fell from early in the crisis (λ(*t*) = 0.542, 0.657 in weeks 1, 2) to later in the crisis (λ(*t*) = 0.349, 0.399 in week 18,20). This indicates that the influence of spatially related omitted variables fell over this time period.

The fixed effect estimates across jurisdictions shown in Table 6 show some interesting features. Strikingly, at a one percent level (two tailed test), none of the 49 jurisdictions show a statistically significant effect in weeks 1 through 8 relative to the base jurisdiction of the District of Columbia. In week 10 and 11, eight and five of the 49 states did exceed the critical value of 2.58, respectively. Despite the paucity of *t*−values with a large magnitude, a joint likelihood ratio test across all jurisdictions shows in terms of a fixed effects versus a single intercept model, twice the difference in likelihood between SEM-FE and SEM gives values between 84.8 to 199.2 over the 20 weeks. The critical value at one percent would be 74.92 (based on 49 d.f.). Therefore, one cannot reject the hypothesis that the fixed effects are jointly significant. Interestingly, the intercept in Table 6 only differs significantly from zero in 3 out of the 20 weeks.

To obtain some insight into the relative contributions of jurisdictional fixed effects and of demographic exogenous variables, we break apart the overall prediction $\hat{\Delta }\left(t\right)$ in (34) into a fixed effects component $\hat{\Delta }{\left(t\right)}_{FE}$ in (35) and a demographic component $\hat{\Delta }{\left(t\right)}_{D}$ in (36). As is well known, the variance of the sum of two random variables equals the sum of their individual variables along with an interaction term in (37).

$$\begin{array}{c} \hat{\Delta }\left(t\right)=\hat{\Delta }{\left(t\right)}_{FE}+\hat{\Delta }{\left(t\right)}_{D} \end{array}$$ (34)

$$\begin{array}{c} \hat{\Delta }{\left(t\right)}_{FE}=S\hat{\alpha }\left(t\right) \end{array}$$ (35)

$$\begin{array}{c} \hat{\Delta }{\left(t\right)}_{D}=Z\hat{\beta }{\left(t\right)}_{SDEM-FE} \end{array}$$ (36)

$$\begin{array}{cc} \mathrm{var}\left(\hat{\Delta }\left(t\right)\right) & =\mathrm{var}\left(\hat{\Delta }{\left(t\right)}_{FE}\right)+\mathrm{var}\left(\hat{\Delta }{\left(t\right)}_{D}\right)\\ & +2\mathrm{var}{\left(\hat{\Delta }{\left(t\right)}_{FE}\right)}^{1/2}\cdot \mathrm{var}{\left(\hat{\Delta }{\left(t\right)}_{D}\right)}^{1/2}\cdot \mathrm{corr}\left(\hat{\Delta }{\left(t\right)}_{FE},\hat{\Delta }{\left(t\right)}_{D}\right) \end{array}$$ (37)

To make (37) more intuitive, we redefine (37) in terms of *R*^2^(*t*) in (38) with *R*^2^(*t*) for individual components of *R*^2^(*t*)_*FE*_ in (39) and *R*^2^(*t*)_*D*_ in (40). This allows writing the overall *R*^2^(*t*) in terms of the individual components of *R*^2^(*t*)_*FE*_ and *R*^2^(*t*)_*D*_ as well as the correlation between the predictions $\mathrm{corr}(\hat{\Delta }{\left(t\right)}_{FE},\hat{\Delta }{\left(t\right)}_{D})$ in (41).

$$\begin{array}{c} R^{2}\left(t\right)=\frac{\mathrm{var}(\hat{\Delta }\left(t\right))}{\mathrm{var}\left(\Delta (t)\right)} \end{array}$$ (38)

$$\begin{array}{c} R^{2}{\left(t\right)}_{FE}=\frac{\mathrm{var}\left(\hat{\Delta }{\left(t\right)}_{FE}\right)}{\mathrm{var}\left(\Delta (t)\right)} \end{array}$$ (39)

$$\begin{array}{c} R^{2}{\left(t\right)}_{D}=\frac{\mathrm{var}\left(\hat{\Delta }{\left(t\right)}_{D}\right)}{\mathrm{var}\left(\Delta (t)\right)} \end{array}$$ (40)

$$\begin{array}{c} R^{2}\left(t\right)=R^{2}{\left(t\right)}_{FE}+R^{2}{\left(t\right)}_{D}+2\cdot R_{FE}\cdot R_{D}\cdot \mathrm{corr}\left(\hat{\Delta }{\left(t\right)}_{FE},\hat{\Delta }{\left(t\right)}_{D}\right) \end{array}$$ (41)

From (41), when the correlations between the fixed effect and demographic predictions are negative, the sum of the relative variances overstates *R*^2^(*t*). In other words, when correlations are negative, the predictions from fixed effects and demographic variables work partially in opposite directions and thus the overall variance of the prediction is lower than the sum of the parts. When correlations are positive, the predictions from fixed effects and demographic variables work partially in the same directions and thus the overall variance of the prediction is higher than the sum of the parts. Note, when the correlation between the fixed effect predictions and the demographic predictions equals 0, the overall *R*^2^(*t*) just equals the sum of fixed effect *R*^2^(*t*)_*FE*_ and the demographic variable *R*^2^(*t*)_*D*_.

In the context of social distancing, a positive correlation between the fixed effect predictions and the demographic predictions suggests that the more restrictive counties also had individuals reducing their exposure, while the less restrictive counties had individuals taking on relatively more exposure. A negative correlation suggests that the actions of individuals and counties partially cancel.

Table 7 shows the variance of *y* by each week as well as *R*^2^(*t*)_*FE*_, *R*^2^(*t*)_*D*_, *R*^2^(*t*), and the correlation between $\hat{\Delta }{\left(t\right)}_{FE}$ and $\hat{\Delta }{\left(t\right)}_{D}$. Several patterns emerge from Table 7. First, the variance increases by over eight-fold from week 1 to to its peak at week 9, before falling by about 50% by the end of the data in week 20. Second, the overall *R*^2^(*t*) increases from 0.1594 to 0.5685 from week 1 to week 9, before falling to 0.4809 in week 20. Third, the correlation between $\hat{\Delta }{\left(t\right)}_{FE}$ and $\hat{\Delta }{\left(t\right)}_{D}$ is materially negative from weeks 1 through 5, almost zero from weeks 6 to 13, and materially positive from weeks 14 to 20. Notwithstanding their magnitudes, all the correlations, except in weeks 6, 12, and 13, were statistically significant from 0 at the 1% level. Therefore, the additive property of the relative *R*^2^(*t*) components approximately holds from week 6 through 13. Fourth, over weeks 8 through 20 the explanatory power of demographic variables *R*^2^(*t*)_*D*_ rises relative to its values over weeks 1 through 7. Note, during this latter half of the sample, states invoked varying portfolios of non-pharmaceutical interventions to encourage social distancing. These actions, as a well as a national declaration of a state of emergency and increased media coverage of the pandemic, simultaneously increased the salience of the risk of COVID-19. By week 14, more positive correlations between the fixed effect predictions and the demographic variable predictions emerged, which suggests some reinforcing alignment between jurisdictional and individual behavior.

**Table 7:** Variance of predictions of staying at home from state fixed effect, explanatory variables, relative variances, correlation between these, and *R*^2^(*t*).

Week	Var.	Rel. Var.	Rel. Var.	Ratio	Correlation	*R*^2^(*t*)
	Stay at Home	Fixed Effect Predictions	Demo. Predictions	Demo. to FE Var	FE, Demo. Predictions	
1	0.0050	0.1287	0.0419	0.3258	−0.0762	0.1594
2	0.0086	0.1302	0.0233	0.1786	−0.2156	0.1297
3	0.0078	0.1630	0.0157	0.0961	−0.1239	0.1661
4	0.0083	0.1827	0.0349	0.1908	−0.0929	0.2027
5	0.0084	0.1865	0.1352	0.7251	−0.4276	0.1859
6	0.0187	0.1718	0.0647	0.3764	−0.0210	0.2321
7	0.0277	0.1191	0.1799	1.5100	−0.0566	0.2824
8	0.0396	0.1799	0.3610	2.0070	−0.0782	0.5010
9	0.0474	0.1280	0.4184	3.2688	0.0477	0.5685
10	0.0315	0.1473	0.4431	3.0084	−0.0792	0.5499
11	0.0307	0.1594	0.4481	2.8103	−0.0999	0.5542
12	0.0333	0.1311	0.4383	3.3432	−0.0130	0.5632
13	0.0345	0.1168	0.4303	3.6850	0.0256	0.5585
14	0.0313	0.0929	0.4434	4.7717	0.1101	0.5810
15	0.0302	0.0969	0.4145	4.2775	0.1805	0.5837
16	0.0293	0.1045	0.4261	4.0780	0.1108	0.5773
17	0.0275	0.0948	0.3798	4.0069	0.1909	0.5470
18	0.0255	0.0861	0.3870	4.4929	0.1720	0.5359
19	0.0258	0.1009	0.3505	3.4725	0.1820	0.5200
20	0.0234	0.0883	0.3318	3.7591	0.1778	0.4809

We now examine the potential for spatial clustering of social distances. Table 8 shows the week-by-week cross-sectional spatial autoregressive coefficients for the change in social distancing (Δ(*t*)), the overall predicted change in social distancing ($\hat{\Delta }\left(t\right)$), the fixed effect predictions (FE), the demographic variable predictions (D), and the regression residuals from the SEM fixed effect model ($\hat{r}$). The overall predicted change in social distance displays more spatial autocorrelation than the actual change in social distance. Breaking this down further into the fixed effect and the demographic predictions, the fixed effect predictions show more spatial autocorrelation than the overall prediction such that each week has an autoregressive coefficient $\hat{\lambda }{\left(t\right)}_{FE}$ of over 0.98. Thus, the fixed effect predictions slowly vary over space. This occurs because the effects appear in blocks for all the counties in the state and only show transitions at borders. The demographic predictions also display substantial spatial autocorrelation from 0.851 to 0.914 and show the potential for peaks and valleys of changes in social distance. For completeness, we present the spatial autoregressive coefficients for the residuals from the SEM model with fixed effects. These are much lower, but still material.

**Table 8:** Autoregressive coefficient for Δ(*t*), overall prediction, FE prediction, demographic prediction, residuals from SEM FE.

Week	$\hat{\lambda }{\left(t\right)}_{\Delta (t)}$	$\hat{\lambda }{\left(t\right)}_{\hat{\Delta }\left(t\right)}$	$\hat{\lambda }{\left(t\right)}_{FE}$	$\hat{\lambda }{\left(t\right)}_{D}$	$\hat{\lambda }{\left(t\right)}_{\hat{r}}$
1	0.727	0.969	0.986	0.914	0.535
2	0.759	0.973	0.989	0.879	0.646
3	0.669	0.977	0.986	0.878	0.380
4	0.718	0.972	0.984	0.890	0.466
5	0.630	0.917	0.987	0.898	0.421
6	0.692	0.967	0.990	0.888	0.421
7	0.664	0.942	0.987	0.909	0.344
8	0.785	0.931	0.987	0.892	0.414
9	0.808	0.930	0.986	0.896	0.351
10	0.780	0.912	0.984	0.880	0.423
11	0.783	0.911	0.984	0.878	0.396
12	0.786	0.913	0.986	0.874	0.389
13	0.806	0.914	0.985	0.879	0.472
14	0.799	0.906	0.986	0.866	0.430
15	0.798	0.905	0.986	0.854	0.383
16	0.793	0.904	0.988	0.856	0.376
17	0.797	0.910	0.983	0.860	0.388
18	0.779	0.910	0.982	0.868	0.339
19	0.780	0.904	0.984	0.853	0.378
20	0.758	0.901	0.982	0.851	0.368

In summary, the change in social distancing shows substantial spatial dependence which leads to hotspots (peaks) and coldspots (valleys) in social distancing behavior and that the fixed effect predictions are too smooth (spatially autocorrelated) to account for the variation. Of the various components forming the prediction, the demographic predictions come closest to matching the spatial character of actual changes in social distance.

## Specification robustness

In this section we examine the robustness of the findings to alternative formulations of the model. Specifically, in the following five subsections, we (a) fit SEM without fixed effects; (b) calculate marginal effects from SDM with fixed effects; (c) estimate SEM-FE employing an alternative measure of social distance; (d) examine the correlations from the marginal effects of the demographic variables from various models using different *W* and with different dependent variables; and (e) examine the sensitivity of the results from employing alternative specifications of the explanatory variables.

### SEM without fixed effects

In the specification search section of our paper, we computed the BIC from SEM, SAR, SDEM, and SDM with and without fixed effects. In 14 out of the 20 weeks SEM without fixed effects achieved the lowest BIC in 14 out of the 20 weeks. Nonetheless, we selected SEM with fixed effects as the base model because theoretical considerations indicated that the model should have fixed effects. In this subsection, we explore the performance of SEM without fixed effects. Specfically, we fit (27) using maximum likelihood. The results appear in Table 9. The signs and magnitudes for the coefficients seem similar to those for the base model, and we will take up a more detailed comparison in a later subsection. However, we note that the autoregressive coefficient λ(*t*) has increased substantially from the base model estimates and now ranges between 0.637 to 0.766 versus 0.349 to 0.657 for the base regression in Table 5. This points to spatial error dependence as a more parsimonious substitute for fixed effects across jurisdictions.

**Table 9:** ‘stay-at-home’ SEM estimates without state fixed effects.

Week	Con.	Pop	Child	HS	White	Inc	λ(*t*)
1	0.392	−0.006	−0.024	−0.051	−0.008	−0.003	0.718
	2.126	−5.695	−3.252	−1.833	−1.297	−0.294	27.517
2	0.798	−0.005	−0.009	−0.143	0.005	−0.000	0.766
	3.391	−3.597	−0.945	−4.019	0.726	−0.043	31.984
3	0.513	−0.003	−0.007	−0.111	0.002	0.005	0.675
	2.130	−1.808	−0.748	−3.036	0.303	0.463	23.846
4	0.533	−0.006	0.008	−0.158	0.014	0.013	0.723
	2.222	−4.374	0.882	−4.348	1.885	1.131	27.129
5	0.922	−0.011	0.031	−0.291	0.052	0.017	0.686
	3.701	−7.307	3.139	−7.667	6.538	1.394	23.160
6	−0.326	0.017	0.014	−0.084	0.023	0.038	0.705
	−0.909	7.791	0.995	−1.553	1.970	2.214	24.997
7	−0.574	0.032	0.076	−0.138	0.040	0.055	0.625
	−1.340	12.320	4.397	−2.128	3.025	2.686	19.955
8	−0.990	0.034	0.120	−0.420	0.097	0.201	0.749
	−2.281	12.141	6.834	−6.367	7.007	9.389	26.298
9	−1.472	0.045	0.123	−0.405	0.110	0.234	0.709
	−3.337	15.394	6.942	−6.022	7.814	10.881	23.252
10	0.452	0.028	0.103	−0.640	0.080	0.191	0.741
	1.229	11.824	6.980	−11.378	6.768	10.573	26.406
11	0.822	0.028	0.096	−0.687	0.082	0.179	0.752
	2.292	11.882	6.618	−12.465	7.091	10.163	27.082
12	1.042	0.028	0.088	−0.750	0.082	0.183	0.726
	2.809	11.822	5.885	−13.153	6.891	10.097	25.462
13	1.030	0.033	0.084	−0.717	0.070	0.168	0.736
	2.803	13.482	5.685	−12.667	5.950	9.344	25.076
14	1.857	0.032	0.072	−0.816	0.031	0.148	0.691
	5.351	13.861	5.193	−15.251	2.872	8.816	22.699
15	2.653	0.029	0.048	−0.899	0.019	0.123	0.687
	7.744	13.241	3.507	−17.043	1.785	7.398	23.357
16	2.521	0.028	0.063	−0.884	0.013	0.123	0.689
	7.373	12.879	4.618	−16.787	1.208	7.433	23.707
17	2.249	0.029	0.053	−0.771	−0.012	0.110	0.689
	6.700	13.388	3.905	−14.955	−1.144	6.787	23.400
18	1.988	0.030	0.052	−0.708	−0.008	0.104	0.637
	6.041	14.172	3.955	−14.017	−0.772	6.561	20.190
19	2.599	0.026	0.063	−0.785	−0.028	0.080	0.674
	7.743	12.673	4.694	−15.217	−2.639	4.945	23.002
20	2.626	0.026	0.035	−0.741	−0.019	0.069	0.648
	7.887	12.851	2.604	−14.478	−1.774	4.275	21.001

### SDM

The SDM with fixed effects often resulted in the highest likelihood across weeks. As discussed in the model section of our paper, the SDM in (33) results in marginal effects that do not equal parameter estimates. Accordingly, we estimated (33) and computed the direct and indirect average marginal effects which appear in Tables 10 and 11. We omit the estimates of fixed effects as these were not significant at the one percent level. As a casual summary, the estimated direct effects from the SDM with fixed effects match closely the estimates of SEM with fixed effects (estimates are marginal effects for the SEM). As before, we will takeup a more detailed comparison between the base regression, SEM without fixed effects, and the direct effects from SDM with fixed effects in a later subsection.

**Table 10:** ‘stay-at-home’ SDM direct effects (with state fixed effects—Not shown).

Week	Pop	Child	HS	White	Inc
1	−0.007	−0.017	−0.071	−0.010	−0.003
	−5.778	−2.231	−2.464	−1.512	−0.355
2	−0.007	−0.005	−0.160	−0.005	0.009
	−4.279	−0.524	−4.233	−0.681	0.762
3	−0.003	0.001	−0.139	−0.001	0.001
	−2.077	0.117	−3.573	−0.112	0.108
4	−0.007	0.016	−0.168	0.016	0.013
	−4.278	1.609	−4.570	1.998	1.090
5	−0.015	0.039	−0.349	0.054	0.013
	−8.526	3.823	−8.993	6.395	1.002
6	0.017	0.019	−0.094	0.024	0.056
	7.128	1.263	−1.681	1.879	2.974
7	0.032	0.065	−0.109	0.020	0.096
	11.159	3.700	−1.637	1.355	4.368
8	0.030	0.117	−0.445	0.079	0.226
	10.141	7.037	−6.882	5.290	10.434
9	0.040	0.125	−0.454	0.091	0.257
	13.398	7.124	−6.664	5.980	11.750
10	0.027	0.096	−0.637	0.070	0.212
	10.921	6.319	−11.227	5.538	11.186
11	0.025	0.096	−0.712	0.074	0.193
	10.317	6.566	−12.970	6.136	10.730
12	0.025	0.092	−0.780	0.067	0.196
	9.744	6.021	−13.620	5.380	10.066
13	0.029	0.090	−0.768	0.061	0.176
	11.949	6.134	−14.089	4.985	9.629
14	0.027	0.083	−0.872	0.021	0.149
	10.952	5.560	−16.662	1.724	8.490
15	0.026	0.062	−0.949	0.015	0.110
	11.587	4.579	−17.700	1.318	6.331
16	0.025	0.071	−0.918	0.006	0.115
	10.766	5.104	−16.626	0.500	6.295
17	0.026	0.067	−0.819	−0.016	0.099
	11.337	4.864	−15.923	−1.376	5.552
18	0.028	0.066	−0.746	0.002	0.084
	12.486	4.861	−14.450	0.185	5.086
19	0.026	0.076	−0.814	−0.013	0.056
	11.390	5.534	−15.510	−1.170	3.380
20	0.027	0.046	−0.749	−0.005	0.049
	11.594	3.359	−14.636	−0.453	2.861

**Table 11:** ‘stay-at-home’ SDM indirect effects (with state fixed effects—Not shown).

Week	Pop	Child	HS	White	Inc
1	−0.010	0.006	−0.042	0.019	0.008
	−2.895	0.233	−0.370	1.151	0.273
2	−0.010	0.002	−0.058	0.087	−0.009
	−2.134	0.055	−0.341	3.549	−0.215
3	−0.011	0.003	−0.155	0.014	−0.017
	−2.956	0.108	−1.263	0.810	−0.587
4	−0.010	−0.015	−0.085	−0.022	−0.001
	−2.438	−0.499	−0.653	−1.136	−0.034
5	0.003	−0.003	−0.040	0.013	−0.035
	0.719	−0.112	−0.311	0.667	−1.053
6	−0.020	0.008	0.115	−0.061	0.007
	−3.553	0.199	0.619	−2.181	0.161
7	−0.014	0.019	−0.199	−0.035	−0.051
	−2.059	0.362	−0.897	−1.086	−0.925
8	0.011	0.046	−0.258	−0.071	−0.173
	1.654	0.918	−1.185	−2.258	−3.218
9	0.017	0.017	−0.178	−0.052	−0.177
	2.674	0.338	−0.828	−1.691	−3.308
10	0.011	0.019	−0.215	−0.038	−0.187
	1.902	0.447	−1.143	−1.397	−3.961
11	0.010	0.050	−0.239	−0.014	−0.177
	1.743	1.080	−1.272	−0.490	−3.917
12	0.003	0.036	−0.263	−0.029	−0.139
	0.429	0.827	−1.442	−1.051	−3.073
13	0.012	0.039	−0.307	−0.014	−0.178
	1.954	0.827	−1.562	−0.469	−3.750
14	0.006	−0.012	−0.204	−0.011	−0.102
	1.149	−0.282	−1.100	−0.413	−2.252
15	0.001	0.014	−0.080	0.019	−0.049
	0.172	0.344	−0.470	0.733	−1.138
16	0.002	−0.003	−0.050	−0.020	−0.029
	0.475	−0.085	−0.309	−0.785	−0.720
17	−0.001	0.035	−0.456	0.045	−0.117
	−0.189	0.872	−2.662	1.761	−2.700
18	0.007	−0.020	−0.185	−0.020	−0.079
	1.322	−0.538	−1.135	−0.847	−2.044
19	−0.004	0.032	−0.340	−0.023	−0.068
	−0.641	0.785	−1.948	−0.876	−1.570
20	−0.010	0.037	−0.228	−0.006	−0.043
	−1.960	0.947	−1.359	−0.232	−1.032

### Different measure of social distance

We now turn to an alternative measure of social distance. We examine median home time as opposed to the previous analysis of percent of time spent at home. Table 12 shows the percentiles of this variable by week. Like percent of time spent at home, median home time started at a lower level, reached a peak around week 12, and then declined.

**Table 12:** Percentiles of median home time by week.

Week	Min	5th	10th	25th	50th	75th	90th	95th	Max
1	18.00	65.45	67.74	70.34	72.46	74.77	77.29	79.20	97.57
2	27.71	65.43	67.56	69.49	71.49	73.51	76.01	78.19	98.29
3	6.29	67.89	69.48	71.62	74.13	76.79	79.37	81.34	99.00
4	21.00	63.21	65.86	68.50	70.64	72.91	75.30	76.97	97.71
5	15.86	62.78	65.58	68.16	70.36	72.62	74.97	76.92	97.86
6	29.71	62.51	65.69	68.70	70.94	73.18	75.70	77.48	100.00
7	12.00	50.38	55.53	60.68	64.50	67.24	69.52	71.13	96.43
8	9.57	50.00	56.87	63.37	67.38	70.41	72.96	74.78	98.00
9	1.14	59.87	65.92	72.74	77.35	80.92	84.53	86.85	99.14
10	10.19	61.63	68.83	76.03	80.96	85.41	88.95	91.14	99.14
11	17.71	76.79	80.17	84.38	87.75	90.80	93.44	95.11	99.14
12	23.71	79.49	82.94	86.68	89.69	92.48	94.95	96.28	99.93
13	7.14	77.38	81.17	85.36	88.86	91.97	94.55	96.04	100.00
14	9.71	73.23	77.42	81.64	85.36	89.05	92.21	94.15	99.71
15	28.57	74.87	77.57	80.96	84.36	88.03	91.56	93.51	99.18
16	26.14	75.54	78.13	81.40	85.23	89.22	92.52	94.43	99.43
17	23.36	72.77	75.45	78.97	82.66	86.43	90.59	93.01	99.57
18	3.57	69.71	72.73	76.09	79.87	83.91	88.37	91.18	99.57
19	22.67	69.02	72.38	76.00	79.28	82.82	87.25	89.88	98.57
20	9.43	68.15	70.77	73.93	76.83	80.62	85.85	88.62	98.43
21	35.46	70.34	72.70	75.32	78.29	82.17	86.79	89.21	99.36

Table 13 provides estimates of SEM with fixed effects for the exogenous variables. The results qualitatively match those from estimating SEM with fixed effects when using the percentage stayed at home dependent variable. Namely, Pop, Child, and Income have a positive effect on social distancing while HS has a negative effect on social distancing. The White variable shows some differences from the previous analysis as it goes from significant and positive in the early weeks but becomes significant and negative in the last two weeks. We will go into a further analysis of the similarity of the estimates from different methods, different *W*, and the two dependent variables in the following section.

**Table 13:** Median home time SEM estimates (with state fixed effects—Not shown).

Week	Pop	Child	HS	White	Inc	λ(*t*)
1	−0.004	−0.012	−0.075	−0.001	−0.015	0.185
	−5.711	−2.249	−3.827	−0.298	−2.524	3.680
2	−0.005	−0.019	−0.126	−0.016	−0.033	0.359
	−3.982	−2.533	−4.362	−2.737	−3.709	7.667
3	−0.000	−0.004	−0.118	0.013	−0.031	0.131
	−0.104	−0.525	−3.000	2.359	−3.572	2.528
4	−0.000	0.014	−0.091	0.012	−0.015	0.019
	−0.321	1.757	−2.908	2.008	−1.630	0.355
5	−0.004	0.025	−0.183	0.034	−0.027	0.058
	−3.409	3.090	−5.932	5.817	−2.979	1.101
6	0.024	−0.001	0.075	0.024	0.014	0.177
	11.050	−0.055	1.394	2.315	0.870	3.587
7	0.026	0.057	0.127	0.034	0.025	0.187
	10.530	3.556	2.056	2.838	1.352	3.742
8	0.026	0.028	0.072	0.027	0.080	−0.025
	9.744	1.598	1.054	2.121	4.061	−0.457
9	0.021	0.054	0.074	0.050	0.099	0.086
	9.056	3.524	1.251	4.465	5.681	1.651
10	0.006	0.004	−0.054	0.011	0.078	0.159
	4.057	0.397	−1.405	1.461	6.808	3.102
11	0.001	0.004	−0.102	0.018	0.062	0.113
	0.876	0.388	−2.655	2.379	5.478	2.119
12	0.002	0.008	−0.099	0.029	0.077	0.111
	1.515	0.764	−2.533	3.858	6.670	2.129
13	0.004	0.035	−0.184	0.032	0.056	0.149
	2.097	2.924	−3.999	3.593	4.162	2.804
14	0.006	0.013	−0.257	0.001	0.053	0.182
	3.902	1.352	−7.130	0.080	4.976	3.535
15	0.005	0.014	−0.400	−0.002	0.024	0.242
	3.704	1.558	−11.508	−0.308	2.320	4.926
16	0.008	0.017	−0.345	−0.003	0.038	0.219
	5.664	1.764	−9.085	−0.411	3.342	4.344
17	0.012	0.013	−0.283	−0.021	0.046	0.373
	6.823	1.180	−6.420	−2.334	3.421	7.873
18	0.015	0.025	−0.183	−0.004	0.040	0.259
	9.860	2.437	−4.712	−0.489	3.401	5.263
19	0.012	0.046	−0.250	−0.025	0.020	0.164
	6.867	4.006	−5.655	−2.907	1.541	3.149
20	0.008	0.012	−0.308	−0.024	0.011	0.164
	5.659	1.269	−8.687	−3.541	1.078	3.222

### Correlations of marginal effects across models

We now compare the base case SEM with fixed effects using 15 nearest neighbors, direct effects from the SDM with fixed effects using 15 nearest neighbors, SEM without fixed effects (intercept only) using 15 nearest neighbors, and the direct effects from SDEM with fixed effects using 15 nearest neighbors, direct effects from SAR with fixed effects using 15 nearest neighbors, SEM with fixed effects using *W* based on 10 nearest neighbors, SEM with fixed effects using *W* based on 20 nearest neighbors, and using the alternative dependent variable of median home time estimated via SEM with fixed effects and 15 nearest neighbors. We do this for four variables—Child, HS, White, and Income. To avoid a table, we did not do this with Pop, but the results are similar.

We compute the correlations between the methods across weeks and the results appear in Table 14. The upper triangle gives correlations among estimated coefficients across specifications for the Child variable and the lower triangle does the same for the HS variable. We see that the various methods appear to substantially agree with each other, with the minimum correlation across methods of 0.59 for the Child variable estimated by SAR with fixed effects using log of percent home and SEM with fixed effects using log of median home time. The largest correlations were 1.0000 for the HS variable estimated with SEM with fixed effects and SDM with fixed effects.

**Table 14:** Correlations among model effects: Child in upper triangle, HS in lower triangle.

Child → HS ↓	SEM FE	SDM Direct	SEM Con.	SDEM FE	SAR FE	SEM FE 10 NN	SEM FE 20 NN	SEM FE Home Time
SEM FE	.	0.9987	0.9919	0.9991	0.9968	0.9998	0.9999	0.6041
SDM Direct	0.9994	.	0.9878	0.9999	0.9962	0.9983	0.9984	0.6133
SEM Constant	0.9988	0.9978	.	0.9891	0.9844	0.9921	0.9924	0.6123
SDEM FE	0.9995	1.0000	0.9980	.	0.9958	0.9986	0.9989	0.6140
SAR FE	0.9983	0.9977	0.9974	0.9978	.	0.9972	0.9958	0.5918
SEM FE 10 NN	0.9999	0.9992	0.9986	0.9993	0.9975	.	0.9996	0.5987
SEM FE 20 NN	1.0000	0.9995	0.9987	0.9996	0.9982	0.9998	.	0.6013
Home Time SEM	0.6938	0.6892	0.6896	0.6904	0.6567	0.7034	0.6934	.

We perform a similar exercise when examining the White and Income variables in Table 15. The lowest correlation for White of 0.7436 is between SEM with fixed effects using log of median home time as the dependent variable and SAR with fixed effects when using log of percent home as the dependent variable. For income the lowest correlation is 0.9473 between SAR with fixed effects when using log of percent home as the dependent variable and SEM with fixed effects when using log of median home time as the dependent variable. Overall, the correlations between the estimated coefficients are quite high.

**Table 15:** Correlations among model effects: White in upper triangle, income in lower triangle.

White → Income ↓	SEM FE	SDM Direct	SEM Con.	SDEM FE	SAR FE	SEM FE 10 NN	SEM FE 20 NN	SEM FE Home Time
SEM FE	.	0.9908	0.9849	0.9920	0.9929	0.9986	0.9997	0.8007
SDM Direct	0.9989	.	0.9904	0.9998	0.9737	0.9849	0.9893	0.8259
SEM Constant	0.9885	0.9848	.	0.9900	0.9695	0.9773	0.9836	0.8204
SDEM FE	0.9989	0.9999	0.9852	.	0.9764	0.9867	0.9903	0.8195
SAR FE	0.9898	0.9901	0.9629	0.9905	.	0.9957	0.9921	0.7436
SEM FE 10 NN	0.9998	0.9989	0.9862	0.9989	0.9909	.	0.9983	0.7843
SEM FE 20 NN	0.9999	0.9990	0.9871	0.9989	0.9903	0.9999	.	0.8045
Home Time SEM	0.9703	0.9685	0.9705	0.9688	0.9473	0.9698	0.9700	.

The high correlations among the marginal effects across the models provides indirect support for the basic specification. Models that differ by specfications of the disturbances should yield very similar estimates for large data sets under correct specification of the explanatory variable part of the model. To formalize this intuition, [35] used this to propose a Hausman test for the spatial error model relative to OLS. To the degree that the SDM and SEM models showed relatively weak explanatory power of indirect effects, these models also come closer to being error models, and thus the agreement between their results and those from other models should not be surprising. In addition, [36] showed that small changes in *W* may have less effect than commonly thought because a 20 nearest neighbor weight matrix and a 19 nearest neighbor weight matrix have 95% of the same positive elements when basing the choice of neighbors on the same metric. For two weight matrices *W*_*a*_ with *m*_*a*_ neighbors and *W*_*b*_ with *m*_*b*_ neighbors where *m*_*a*_ ≤ *m*_*b*_ and *v* ∼ *N*(0, *I*_*n*_), corr(*W*_*a*_ *v*, *W*_*b*_ *v*) = (*m*_*a*_/*m*_*b*_)^1/2^. This also appears to happen here since we obtained virtually the same results from using 10, 15, and 20 nearest neighbors.

### Explanatory variable choice and *R*^2^ decomposition

The base model employed used five variables on population density, age, education, race, and income (all logged). Of course, many other specifications could have been used and this poses the question of the robustness of the decomposition of the fixed effect and demographic predictions. In this subsection we provide an augmented model and examine the resulting decomposition. In the alternative model we augment the base model with (logged) non-black (coded this way to avoid 0), high school education (which differs from HS in the base model because not having a college degree does not imply having a high school degree), household size, percent on social security, percent with insurance, percent of children, and three variables measuring the percent of individuals of age 60 and older. The augmented model provides some alternative proxies for basic demographics. To keep the dimension of the model the same, we took all of the augmented variables, extracted the first five principal components, and repeated the *R*^2^(*t*) decomposition in Table 16. Relative to Table 7, the *R*^2^(*t*) for the principal components model is usually higher by a small amount in the later periods and the relative variance of the demographic to the fixed effect predictions is somewhat higher. The principal components reach their highest relative variance in week 18 at 5.1758 versus 4.7717 for the base model in week 14. As this illustrates, better models have the potential to show that demographic variables may have a greater influence on social distancing behavior relative to the base model. As more demographic variables are added and more optimization of the model occurs, this suggests that the importance of demographic variables will rise relative to fixed effects.

**Table 16:** Variance of predictions of staying at home from state fixed effect, explanatory variables, relative variances, correlation between these, and *R*^2^(*t*).

Week	Var.	Rel. Var.	Rel. Var.	Ratio	Correlation	*R*^2^(*t*)
	Stay at Home	Fixed Effect Predictions	Demo. Predictions	Demo. to FE Var	FE, Demo. Predictions	
1	0.0050	0.1253	0.0445	0.3554	−0.0606	0.1608
2	0.0086	0.1210	0.0196	0.1622	−0.1196	0.1289
3	0.0078	0.1598	0.0150	0.0936	−0.1026	0.1648
4	0.0083	0.1792	0.0309	0.1727	−0.0832	0.1977
5	0.0084	0.1987	0.1419	0.7140	−0.4714	0.1823
6	0.0187	0.1782	0.0626	0.3516	−0.0484	0.2306
7	0.0277	0.1181	0.1797	1.5217	−0.0818	0.2740
8	0.0396	0.1759	0.3549	2.0175	−0.0938	0.4839
9	0.0474	0.1216	0.4141	3.4044	0.0374	0.5525
10	0.0315	0.1546	0.4388	2.8374	−0.1065	0.5379
11	0.0307	0.1711	0.4497	2.6276	−0.1322	0.5474
12	0.0333	0.1422	0.4368	3.0710	−0.0474	0.5553
13	0.0345	0.1170	0.4340	3.7090	0.0123	0.5565
14	0.0313	0.0950	0.4431	4.6636	0.1035	0.5806
15	0.0302	0.1153	0.4187	3.6303	0.1257	0.5892
16	0.0293	0.1060	0.4232	3.9928	0.1098	0.5756
17	0.0275	0.0927	0.3866	4.1730	0.2000	0.5550
18	0.0255	0.0754	0.3902	5.1758	0.2155	0.5396
19	0.0258	0.0878	0.3606	4.1082	0.2343	0.5318
20	0.0234	0.0750	0.3399	4.5340	0.2469	0.4936

## Conclusion

We employed cell phone tracking data to obtain a picture of how stay-at-home behavior evolved during the initial spread of COVID-19 in the United States. Because of simultaneity issues associated with stay-at-home behavior and the spread of the disease, and the problems of decentralized disease measurement in the United States, we posited a reduced form model that uses changes in stay-at-home behavior by county over time as a function of exogenous demographic variables such as population density, households with children, education, race, and income. Due to the contagious nature of COVID-19, we focused on possible spatial aspects of the behavior. We modeled spatial aspects using: (1) changes in social distancing behavior that reduce the influence of omitted variables, spatial or otherwise; (2) fixed effects for each jurisdiction; (3) spatial spillovers as coming from the exogenous demographic variables from surrounding counties; and (4) spatial autoregressive behavior of disturbances from surrounding counties.

Our research produced three main results. First, we found that as the crisis progressed the demographic exogenous variables explained an increasing proportion of the overall variance of behavior. By week 12, demographic exogenous variables explained over three times as much variance as jurisdictional fixed effects; by week 18 demographic variables explained over four times as much variance as jurisdictional fixed effects, before falling to 3.76 times in week 20. Second, over the span of the data, the correlation between fixed effect predictions and demographic variable predictions went from significant and negative in weeks 1–5 to relatively low magnitude correlations in periods 6–13, then finished with significant and positive correlations in periods 14–20. A negative correlation signifies fixed effect predictions and demographic variable predictions partially cancel each other. A zero correlation signifies that the fixed effect predictions and demographic variable predictions operate independently. A positive correlation signifies that fixed effects predictions and demographic variable predictions reinforce or complement each other. In other words, states with positive fixed effects also had larger amounts of social distancing based on demographic variables. Third, we found that observed social distancing and the demographic variables showed high levels of spatial autocorrelation or clustering which result in hotspots or peaks as well as coldspots or valleys in social distances. In summary: (1) fixed effects explain less of the variance in social distancing behavior relative to demographic variables; (2) correlations exist between fixed effect predictions and demographic variable predictions that result in partial cancellation or reinforcement in the overall predicted social distances; and (3) observed social distances and demographic variables display high levels of spatial autocorrelation or clustering.

What are the implications of our results? First, the strong tendency of social distances to cluster has a number of ramifications. Clustering of low social distance counties could show increasing levels of disease while clusters or coldspots of high social distance counties could show decreasing levels of disease. Even if this leads to an aggregate decrease over some period, having clusters of low social distance counties with increasing incidence of disease may impede economic recovery, even in clusters of high social distance counties, because of the potential for contagion between low and high social distance county clusters. Second, although relatively severe restrictions have worked in various places, in the absence of binding jurisdictional restrictions, persuading individuals to increase their social distancing voluntarily may provide a lower cost means to reducing disease incidence. Third, such persuasion may also improve acceptance of restrictions and result in a positive correlation between individual and jurisdictional actions that reinforces the amount of social distancing. This potentiation or reinforcement provides additional returns to NPIs that raise actual or effective (e.g., masks) social distancing.

## Supporting information

S1 File (PDF)

## References

[1] Kennedy P. A guide to econometrics. Third Ed, Cambridge: MIT Press; 1982.

[2] Carroll RJ, Ruppert D, Stefanski LA, Crainiceanu CM. Measurement error in nonlinear models, a modern perspective. Boca Raton: Chapman and Hall 2006.

[3] Lam NS, Arenas H, Pace RK, LeSage JP, Campanella R. Predictors of business return in New Orleans after Hurricane Katrina. PloS One. 2012;7(10): e47935 10.1371/journal.pone.0047935 23133530 PMC3480509

[4] Lam NS, Pace RK, Campanella R, Lesage JP, Arenas H. Business return in New Orleans: decision making amid post-Katrina uncertainty. PloS One. 2009;4(8): e6765 10.1371/journal.pone.0006765 19707547 PMC2727799

[5] Stock JH. Data gaps and the policy response to the novel coronavirus. NBER [Preprint]. 2020 NBER 26902 [posted May 2020; cited 25 May 2020]. Available from: https://www.nber.org/papers/w26902.

[6] Avery, C, Bossert W, Clark A, Ellison G, Ellison S. Policy implications of models of the spread of coronavirus: perspectives and opportunities for economists. NBER [Preprint]. 2020 NBER 27007 [posted April 2020; cited 25 May 2020]. Available from: https://www.nber.org/papers/w27007.

[7] Allcot H, Boxell L, Conway J, Gentzkow M, Thaler M, Yang D. Polarization and public health: partisan differences in social distancing during the coronavirus pandemic. SSRN [Preprint]. 2020 SSRN 3570274 [posted 7 Apr 2020; revised 19 May 2020; cited 25 May 2020]. Available from: https://ssrn.com/abstract=3570274. 10.1016/j.jpubeco.2020.104254 PMC7409721 32836504

[8] Aum, S, Lee SY, Shin Y. Inequality of fear and self-quarantine: is there a trade-off between GDP and public health? NBER [Preprint]. 2020 NBER 27100 [posted May 2020; cited 25 May 2020]. Available from: https://www.nber.org/papers/w27100.

[9] Barrios J, Hochberg Y. Risk perception through the lens of politics in the time of the COVID-19 pandemic. NBER [Preprint]. 2020 NBER 27008 [posted April 2020; cited 25 May 2020]. Available from: https://www.nber.org/papers/w27008.

[10] Brzezinski, A, Kecht V, Van Dijcke D, Wright A. Belief in science influences physical distancing in response to COVID-19 lockdown policies. SSRN [Preprint]. 2020 SSRN 3587990 [posted 4 May 2020; cited 25 May 2020]. Available from https://ssrn.com/abstract=3587990.

[11] McFadden S, Malik A, Aguolo O, Willebrand K, Omer S. Perceptions of the adult US population regarding the novel coronavirus outbreak. PloS One. 2020;15(4): e0231808 10.1371/journal.pone.0231808 32302370 PMC7164638

[12] Painter M, Qiu T. Political beliefs affect compliance with COVID-19 social distancing orders. SSRN [Preprint]. 2020 SSRN 3569098 [posted 6 April 2020; cited 25 May 2020]. Available from: https://ssrn.com/abstract=3569098.

[13] Rampini A. Sequential lifting of COVID-19 interventions with population heterogeneity. NBER [Preprint]. 2020 NBER 27063 [posted April 2020; cited 25 May 2020]. Available from: https://www.nber.org/papers/w27063.

[14] Acemoglu D, Chernozhukov V, Werning I, Whinston M. A multi-risk SIR model with optimally targeted lockdown. NBER [Preprint]. 2020 NBER 27102 [posted May 2020; cited 25 May 2020]. Available from: https://www.nber.org/papers/w27102.

[15] Anderson, M, Maclean J, Pesko M, Simon K. Effect of a federal paid sick leave mandate on working and staying at home: evidence from cellular device data. NBER [Preprint]. 2020 NBER 27138 [posted May 2020; cited 25 May 2020]. Available from: https://www.nber.org/papers/w27138.

[16] Baker MG, Peckham TK, Seixas NS. Estimating the burden of United States workers exposed to infection or disease: a key factor in containing risk of COVID-19 infection. PLoS One. 2020;15(4): e0232452 10.1371/journal.pone.0232452. 32343747 PMC7188235

[17] Chiou L, Tucker C. Social distancing, internet access and inequality. NBER [Preprint]. 2020 NBER 26982 [posted April 2020; cited 25 May 2020]. Available from: https://www.nber.org/papers/w26982.

[18] Mongey S, Pilossoph L, Weinberg A. Which workers bear the burden of social distancing policies? NBER [Preprint]. 2020 NBER 27085 [posted May 2020; cited 25 May 2020]. Available from: https://www.nber.org/papers/w27085.

[19] Fernández-Villaverde J, Jones C. Estimating and simulating a SIRD model of COVID-19 for many countries, states, and cities. NBER [Preprint]. 2020 NBER 27128 [posted May 2020; cited 25 May 2020]. Available from: https://www.nber.org/papers/w27128. 10.1016/j.jedc.2022.104318 PMC8799324 35125563

[20] Raifman J, Nocka K, Jones D, Bor J, Lipson S, Jay J, et al COVID-19 US state policy database. 2020 [cited 17 July 2020]. Available at: www.tinyurl.com/statepolicies.

[21] Cheng M, Papenburg J, Desjardins M, Kanjilal S, Quach C, Libman M, et al Diagnostic testing for severe acute respiratory syndrome–related Coronavirus 2. Annals of Internal Medicine. 2020;172(11): 726–734. 10.7326/M20-1301 32282894 PMC7170415

[22] Sharfstein J, Becker S, Mello M. Diagnostic testing for the novel Coronavirus. JAMA. 2020;323(15):1437–1438. 10.1001/jama.2020.3864 32150622

[23] Davidson R, MacKinnon J. Estimation and inference in econometrics. New York: Oxford University Press 1993.

[24] Christ C. The Cowles commission’s contributions to econometrics at Chicago, 1939–1955. Journal of Economic Literature. 1994;32(1): 30–59.

[25] Bavaud F. Models for spatial weights: a systematic look. Geographical Analysis. 1998;30: 153–171. 10.1111/j.1538-4632.1998.tb00394.x

[26] LeSage JP, Pace RK. Introduction to spatial econometrics. Boca Raton: Taylor and Francis, CRC Press; 2009.

[27] Elhorst JP. Applied spatial econometrics: raising the bar. Spatial Economic Analysis. 2010;5(1): 9–28. 10.1080/17421770903541772

[28] Vega SH, Elhorst JP. The SLX model. Journal of Regional Science. 2015;55(3): 339–363. 10.1111/jors.12188

[29] Guliyev H. Determining the spatial effects of COVID-19 using the spatial panel data model. Spatial statistics, 100443. 2020. Advance online publication. 10.1016/j.spasta.2020.100443. PMC7139267 32292691

[30] Krisztin T, Piribauer P, Wögerer M. The spatial econometrics of the coronavirus pandemic. 2020 Working paper [posted April 2020; cited 25 May 2020]. Available from: https://www.researchgate.net/publication/340620046_The_spatial_econometrics_of_the_coronavirus_pandemic. 10.1007/s12076-020-00254-1 PMC7395580 33269031

[31] Ord JK. Estimation methods for models of spatial interaction. Journal of the American Statistical Association. 1975;70: 120–126. 10.1080/01621459.1975.10480272

[32] Schwarz G. Estimating the dimension of a model. The annals of statistics. 1978;6(2): 461–464. 10.1214/aos/1176344136

[33] Akaike H. A new look at the statistical model identification. IEEE Transactions on Automatic Control. 1974;19(6): 716–723. 10.1109/TAC.1974.1100705

[34] Bozdogan H. Model selection and Akaike’s information criterion (AIC): the general theory and its analytical extensions. Psychometrika. 1987;52(3): 345–370. 10.1007/BF02294361

[35] Pace RK, LeSage JP. A spatial Hausman test. Economics Letters. 2008;101(3): 282–284. 10.1016/j.econlet.2008.09.003

[36] LeSage JP, Pace RK. The biggest myth in spatial econometrics. Econometrics. 2012;2(4): 217–249. 10.3390/econometrics2040217

