# Supplementary material for: Demographic, jurisdictional, and spatial effects on social distancing in the United States during the COVID-19 pandemic
Source: PLoS One. 2020 Sep 22;15(9):e0239572. doi: 10.1371/journal.pone.0239572 (PMC7508394; doi:10.1371/journal.pone.0239572)
Supplement: S1 File — (PDF) [file pone.0239572.s001.pdf]

Supporting Information S1

Ethics Statement

The following ethics statement applies to the empirical study in "Demographic, jurisdictional, and spatial effects on social distancing in the United States during the COVID-19 pandemic" that uses human subjects. Specifically, the data reveals the movement of human subjects using cell phone ping records to track the movement of the device user.

All data was anonymized and aggregated to a sufficiently high level by the data owner, SafeGraph, before it was made available to the researchers. Researchers may access the data from SafeGraph by requesting access at their COVID-19 Data Consortium site: https://www.safegraph.com/covid-19-data-consortium.
